# Untargeted Analysis of *Lemna minor* Metabolites: Workflow and Prioritization Strategy Comparing Highly Confident Features between Different Mass Spectrometers

**DOI:** 10.3390/metabo11120832

**Published:** 2021-12-02

**Authors:** Rofida Wahman, Stefan Moser, Stefan Bieber, Catarina Cruzeiro, Peter Schröder, August Gilg, Frank Lesske, Thomas Letzel

**Affiliations:** 1 Chair of Urban Water Systems Engineering, Technical University of Munich, Am Coulombwall 3, 85748 Garching, Germany; rofida.wahman@tum.de; 2 Pharmacognosy Department, Faculty of Pharmacy, Assiut University, 71526 Assiut, Egypt; 3 Stefan Moser Process Optimization, Weberweg 3, 83131 Nußdorf am Inn, Germany; info@stefan-moser.com; 4 Analytisches Forschungsinstitut für Non-Target Screening GmbH (AFIN-TS), Am Mittleren Moos 48, 86167 Augsburg, Germany; s.bieber@afin-ts.de; 5 Research Unit Comparative Microbiome Analysis, German Research Center for Environmental Health, Helmholtz Centrum Munich, Ingolstädter Strasse 1, 85764 Neuherberg, Germany; catarina.cruzeiro@helmholtz-muenchen.de (C.C.); peter.schroeder@helmholtz-muenchen.de (P.S.); 6 Departement of Bioengineering Sciences, Weihenstephan-Triesdorf University of Applied Sciences, Am Hofgarten 4, Weihenstephan, 85354 Freising, Germany; august.gilg@online.de (A.G.); frank.lesske@hswt.de (F.L.)

**Keywords:** FOR-IDENT, metabolomics, OPLS-DA, PLANT-IDENT, polarity-extended chromatography, quadrupole time-of-flight (QTOF), TOF

## Abstract

Metabolomics approaches provide a vast array of analytical datasets, which require a comprehensive analytical, statistical, and biochemical workflow to reveal changes in metabolic profiles. The biological interpretation of mass spectrometric metabolomics results is still obstructed by the reliable identification of the metabolites as well as annotation and/or classification. In this work, the whole *Lemna minor* (common duckweed) was extracted using various solvents and analyzed utilizing polarity-extended liquid chromatography (reversed-phase liquid chromatography (RPLC)-hydrophilic interaction liquid chromatography (HILIC)) connected to two time-of-flight (TOF) mass spectrometer types, individually. This study (introduces and) discusses three relevant topics for the untargeted workflow: (1) A comparison study of metabolome samples was performed with an untargeted data handling workflow in two different labs with two different mass spectrometers using the same plant material type. (2) A statistical procedure was observed prioritizing significant detected features (dependent and independent of the mass spectrometer using the predictive methodology Orthogonal Partial Least Squares-Discriminant Analysis (OPLS-DA). (3) Relevant features were transferred to a prioritization tool (the FOR-IDENT platform (FI)) and were compared with the implemented compound database PLANT-IDENT (PI). This compound database is filled with relevant compounds of the Lemnaceae, Poaceae, Brassicaceae, and Nymphaceae families according to analytical criteria such as retention time (polarity and LogD (pH 7)) and accurate mass (empirical formula). Thus, an untargeted analysis was performed using the new tool as a prioritization and identification source for a hidden-target screening strategy. Consequently, forty-two compounds (amino acids, vitamins, flavonoids) could be recognized and subsequently validated in *Lemna* metabolic profile using reference standards. The class of flavonoids includes free aglycons and their glycosides. Further, according to our knowledge, the validated flavonoids robinetin and norwogonin were for the first time identified in the *Lemna minor* extracts.

## 1. Introduction

Metabolomics is the approach that deals with the investigation of a biological system (cell, tissue, or organism) by determining its overall metabolite profile at a given time point with the specified set of conditions. The modern development of analytical techniques expanded the coverage of metabolomics in investigations of biological systems. This approach permits remarkable insights into regulation mechanisms as well as the responses to different perturbations. Over the centuries, quantitative (targeted) metabolomics analysis has become a routine application in different fields [1]. In untargeted metabolomics studies, the identification of (unknown) metabolites is the fundamental step to transform analytical data into biological knowledge. Unfortunately, this transformation is still considered the major bottleneck. So far, the number of identified metabolites in untargeted metabolomics studies is in general below 50% [1]. Several analytical techniques are available in the untargeted metabolomics analysis such as liquid chromatography-mass spectrometry (LC-MS) and gas chromatography-mass spectrometry (GC-MS) [2] to analyze a large number of different chemical metabolites classes within one single analysis, respectively. However, the thermal stability of the stationary phase, metabolites, and their derivatives, which might introduce artifacts, limit the metabolome coverage derived by GC-MS. Thus, the usage of LC-MS has expanded rapidly over the past ten years in untargeted metabolomics analysis [3].

In approaches based on liquid chromatography–high-resolution mass spectrometer (LC-HRMS), metabolites can mostly be identified if the retention time (RT), the ion mass, and MS/MS fragment spectrum of each compound are successfully matched with an authentic reference standard measured in the same instrument. However, due to the presence of over 200,000 to 1,000,000 different metabolites in the plant kingdom, this can be a challenging task [4,5]. Especially the identification of highly specific metabolites that might have significant roles in protecting plants against predators or microbial infection is difficult due to the unavailability of reference standards [4,5]. To overcome this challenge, several MS/MS databases have been established to facilitate metabolite annotation, such as spectral databases MassBank (https://massbank.eu/MassBank/ accessed on 30 November 2021), METLIN (http://metlin.scripps.edu/index.php accessed on 30 November 2021), LipidBlast (https://fiehnlab.ucdavis.edu/projects/lipidblast accessed on 30 November 2021), and ReSpect (http://spectra.psc.riken.jp accessed on 30 November 2021). Moreover, plant metabolomics databases have been developed for potential identification such as KNApSAcK (http://kanaya.naist.jp/KNApSAcK/ accessed on 30 November 2021), MetaCyc (http://metacyc.org accessed on 30 November 2021), KEGG (http://www.genome.jp/kegg/ accessed on 30 November 2021), and PRIMe (http://prime.psc.riken.jp/ accessed on 1 Feburary 2021). Hence, the choice of databases has an essential influence on the interpretation of a scientific study. Small- and medium-sized databases might miss relevant metabolites while large databases contain more molecules so unexpected hits may lead to better information. On the other hand, the small, well-defined databases might suggest more meaningful hits compared to searches in large databases, which may propose compounds that are not related to the respective scientific and biological question [4,5,6,7].

The PLANT-IDENT (PI) database (https://water.for-ident.org accessed on 30 November 2021) for example is a specific compound database containing currently 3019 metabolites of the Lemnaceae family in addition to Poaceae, Brassicaceae, and Nymphaeaceae, as well as other families due to the chemotaxonomy filter in addition to analytical characters. This compound database is implemented for rapid search retrieval into the FOR-IDENT (FI) platform (https://water.for-ident.org accessed on 30 November 2021) using batch search for large numbers of compounds. The FI platform ‘translates’ the analytical features with different analytical parameters like retention time (RT), ion mass, (and MS/MS spectrum) via retention time index (RTI) (by calculating the difference between the LogD values calculated with via RTI of the target mixture and the one one is listed in the FI), and accurate mass into logD values, and molecular formula. These parameters are compared with the physicochemical parameters of the metabolites in the compound database PI which gives a prioritizing hit list (with Look at a function in the FI). Therefore, in general, a large number of molecules from extracts can be annotated and prioritized for the study of these plant families without prior focusing on these compounds. This can enhance the potential coverage of indicators in untargeted metabolomics analysis. A remaining challenge for metabolomic analysis is its weak efficiency and the difficulty of comparing results obtained in different laboratories that use different hardware or data processing. Currently, it may well happen that molecules identified in one laboratory may not be replicable to others [6].

Usually, as it is exemplarily shown in [8], the first step toward metabolites identification is extraction. In the next step, there is an efficient chromatographic separation, providing a reproducible, precise, and wide range of polarity separation. A serial coupling of two LC columns with orthogonal polarities extends the separation of compounds from the complex matrix [8,9,10].

This analysis provides a large amount of data, requiring an adequate workflow to process and analyze it. Although an overall view would be more relevant using all mass spectrometric ion polarity information, exclusively positive ionization mode was applied in this study. This workflow reflects a subset of the untargeted screening results but leads also to statistical useable datasets. HRMS, such as quadrupole time-of-flight (Q-TOF) mass spectrometry and quadrupole-orbitrap (Q-Orbitrap) mass spectrometry have become the most widely used MS tools in metabolomics [6,11]. The metabolomics data processing continues to be problematic, especially the metabolite identification process. The associated workflow contains numerous steps, which have to be precise, compatible, and with less effort and time-consuming. All steps required quality control procedures to ensure trustworthy metabolomics analysis outcomes. Starting from peak picking, signal-to-noise threshold detection, and background correction to the different datasets were aligned. The workflow ended with the application of the automated metabolite identification algorithms and the biological interpretation of the data [12,13].

The most common proposed workflow applies to food and nutrition sciences, where metabolomic analysis has early been adopted as an analytical tool [14]. In addition, medical research [15] uses metabolomics analysis to gain new insights into the interactions between drugs and the human body that are correlated with other clinical variables [16]. In addition, metabolomic analysis is considered in environmental sciences as a technique to evaluate the physiological responses of organisms to the effect of drugs, toxic substances, or metabolic disorders, from the initial chemical interaction to the final adverse outcome [15].

*Lemna minor* is a small, free-floating duckweed species. Fast growth, microbial reduction, and high nutrient and metal accumulation potential are the factors that candidate *Lemna* for phytoremediation research [17]. It belongs to the family Lemnaceae, along with four other aquatic genera, containing various chemical constituents such as amino acids, organic acids, sterol, terpenes, and flavonoids [18,19].

This study is divided into three parts according to the following objectives:

(a) A comparison study of metabolome samples was performed with an untargeted data handling workflow in two different labs with two mass spectrometers (TOF and QTOF) using the same plant material type;
(b) Further, the metabolomics data from different mass spectrometers were analyzed and compared with predictive methodology orthogonal partial least squares—discriminant analysis (OPLS-DA). The discrimination method has been adapted to validate the features that were extracted with the workflow. Consequently, the standard statistical methods of metabolomics data investigation were used in the identification of relevant variables (i.e., conditional attributes to each solvent), which related to the discrimination analysis. Three different extracts were systematically analyzed with this workflow;
(c) Furthermore, the plant metabolites identification workflow was described from the theoretical predictions to the final analyses in *Lemna* metabolic profile using reference materials.

The usage of the open-source PI compound database to identify the different metabolites introduces another helpful tool for plant metabolomics. The PI compound database contains plant metabolites that enable and encourages untargeted screening in plant metabolomics.

## 2. Results and Discussion

**Figure 1:**
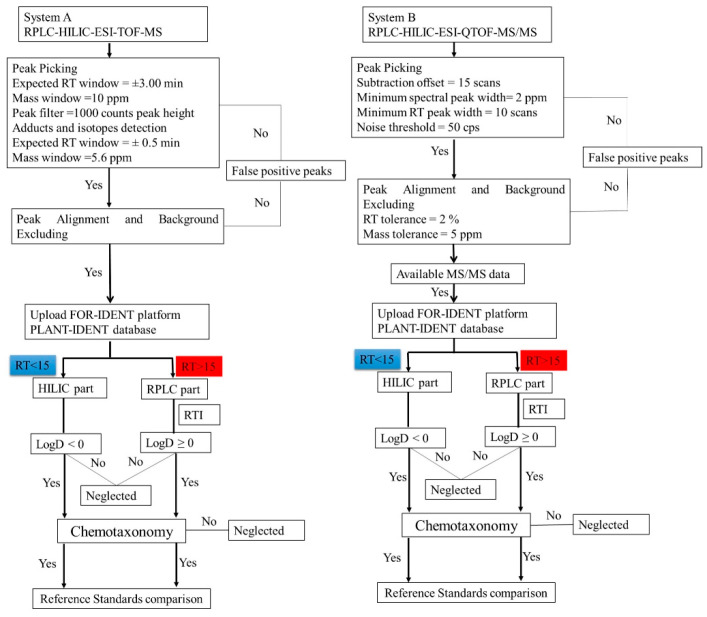
The non-target workflow flowchart (from precursor ion mass to ‘molecular names’) using a RPLC-HILIC-ESI-TOF-MS (system A) and evaluating the analytical data (including MS/MS) obtained with RPLC-HILIC-ESI-QTOF-MS/MS (system B). The parameters of peak picking and alignments were set according to the internal standards. The features were prioritized using the PLANT-IDENT database in the FOR-IDENT platform after various data filtration steps. The workflow of the data analysis with system A was also mentioned in [8,20].

In the current work, the data analysis and comparison of *Lemna* metabolic profiles were performed, as well as a potential identification of the metabolites. The analysis strategy (Figure 1) was conducted on the three extracts (100% MeOH, 50% MeOH, and 100% H_2_O) due to the key findings presented in the recent publication of Wahman et al. [8]. According to the analytical and statistical results, the three solvents could extract *Lemna’s* metabolic profile using the extended chromatographic method. Initially, each extract was analyzed (in triplicate) using a RPLC-HILIC-ESI-TOF-MS (system A) obtaining retention time information and accurate masses for ions. Further, the same extract types were analyzed (each in triplicate) using a RPLC-HILIC-ESI-QTOF-MS/MS (system B). The raw data of both were checked for analytical performance and quality by applying internal standards (details in Material and Methods Section 3.5).

### 2.1. Lemna minor Extracts Untargeted Analysis Using Systems A and B

**Figure 2:**
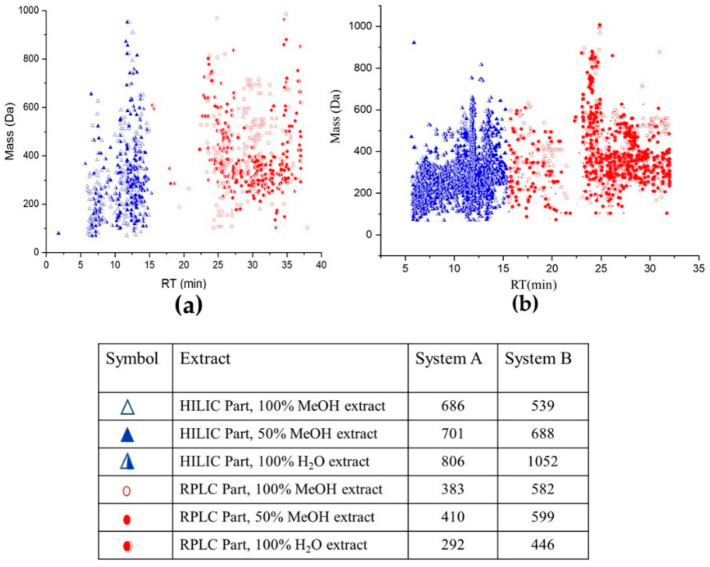
Retention time (RT)—the mass plot of *Lemna minor* metabolic profile extracted with 100% MeOH, 50% MeOH, and 100% H_2_O; using (**a**) system A (single TOF-MS) and (**b**) system B (QTOF-MS/MS). The blue color represents the HILIC eluting metabolites and the red color the RPLC eluting metabolites. The included table reflects the corresponding amount of features.

The obtained data was preprocessed according to the workflow mentioned in Section 3.6 and as shown in Figure 1. A comprehensive strategy was applied to identify metabolites in untargeted *Lemna minor* metabolomics using different labs with different mass spectrometers. Consequently, the mentioned parameters led to 1069, 1111, and 1098 features (in system A in lab 1 (RPLC-HILIC-TOF-MS)) and 1121, 1287, and 1498 features (in system B in lab 2 (RPLC-HILIC-QTOF-MS/MS)) for 100% MeOH, 50% MeOH, and 100% H_2_O extracts, respectively. The RT-mass plots of the obtained features in three solvents (100% MeOH, 50% MeOH, and 100% H_2_O) are plotted in Figure 2 according to their RT (min.) and *m/z*. The dataset was differentiated at 15 min according to the expected features LogD values. The features of the *Lemna minor* metabolic profile eluting from 5 min to 15 min contained the very polar to moderate polar features with expected LogD < 0 (because eluting from the HILIC column) The features eluting later than 15 min represent the nonpolar features with LogD > 0 (because eluting from endcapped C18-RPLC column).

*Lemna minor* metabolic profile was obtained with the single TOF-MS (system A) in Figure 2a and with the QTOF-MS/MS (system B) in Figure 2b with differentiated values for HILIC and RPLC eluting features (see Table in Figure 2). Interestingly, the data showed significant differences in absolute values between the two MS 100% MeOH, 50% MeOH, and 100% H_2_O extracts, respectively (Figure 2). This might be explained by the higher detection sensitivity of the newer system B compared to the older system A, the different data handling processes as well as, the lower number of biological replicates of *Lemna minor*. Another hint for the sensitivity differences can be seen in the retention window from 15 to 23 min. The fundamental differences between both systems in the number of features there are obvious. This section is mainly affected by the start of compounds elution from RPLC and minor changes in the gradient of the mobile phase (details were mentioned in Wahman et al., 2020 [8]).

Further, in our previous study [8,20], the amino acids (phenylalanine, proline, tryptophan, alanine, tyrosine, aspartic acid, isoleucine, serine, and valine) were identified in *L. minor* using system A in the HILIC part [8,19]. Despite being also identified in system B in the HILIC part (Table S1) using target analysis, they were not detected with the common metabolic profile between both systems A and B. This is due to differences in the intensities of the amino acids between the two extracts see Section 3.2.

The overlap of common features between system A and B detected in HILIC and RPLC are small (data not shown). The detailed comparison using multivariate analysis is discussed in the next Section 2.2.

### 2.2. OPLS-DA Analysis of Lemna minor Metabolic Profiles Obtained with Systems A and B

Regarding the untargeted analysis, it is fundamental to assign the common metabolic profile between the different datasets of the same material and/or different materials obtained from the same and/or different laboratories. The untargeted analysis concept depends mainly on prioritizing databases. Thus, the datasets of each system were compared and identification of possible *L. minor* common metabolites was performed via the FOR-IDENT platform and the PLANT-IDENT compound databases.

**Figure 3:**
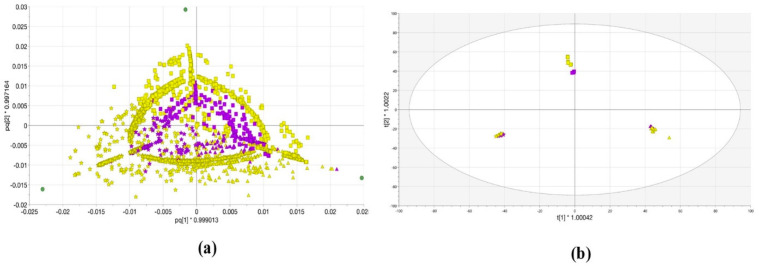
(**a**) The loading plot displays the relation between the different *Lemna minor* samples 100% MeOH (triangular), 50% MeOH (stars), and 100% H_2_O (squares) extracts analyzed with TOF-MS (violet) and QTOF-MS/MS (yellow); (**b**) OPLS-DA score scatter plot of *Lemna minor* samples with 95% confidence limit.

The OPLS-DA calculated differences between the three extracted *Lemna* samples, which colored regarding the analyzed features with single TOF-MS (system A) and QTOF-MS/MS (system B). Hence, coloring the loading score plot (Figure 3a) according to the variables (features) that characterize each extract emphasizes the separation between the mass spectrometer and the dot shape reflects the different extraction solvents measured. Each feature is present in the three extracts but with different intensities (due to their physicochemical conditions and matrix effects in ionization). The OPLS-DA score plot (in Figure 3b) explained 99.2% of the variations in the various extracts (R^2^Y (cum)) with a higher predictive value (Q^2^ (cum) = 0.877). The first component (t1) separated the 100% H_2_O and 50% MeOH extracts in the negative part (of the score plot) and the 100% MeOH extract in the positive part. The orthogonal component (t2) distinguishes significantly between the 100% MeOH and 50% MeOH in the negative and 100% H_2_O positive parts, respectively. Concluding, from the statistical analysis, the *L. minor* metabolic profile has common features between the single TOF and QTOF-MS. In both systems, the different extracts could significantly be discriminated, allowing users to apply this analysis for subsequent *Lemna* metabolite measurements.

### 2.3. OPLS-DA Analysis of Lemna minor 100% MeOH and 100% H_2_O Extracts

The 100% MeOH and 100% H_2_O extracts of *Lemna* using single TOF-MS (system A) were compared with the same extracts obtained using the QTOF-MS/MS (system B) as an example to validate the information transfer between different mass spectrometric systems and/or laboratories.

**Figure 4:**
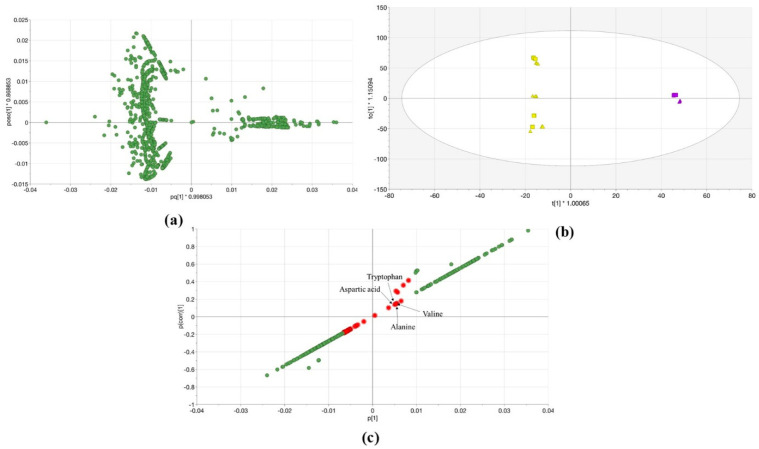
(**a**) The loading plot displays the relation between the different *Lemna minor* samples 100% MeOH and 100% H_2_O extracts analyzed with single TOF-MS (system A) and QTOF-MS/MS (system B); (**b**) OPLS-DA score scatter plot of *Lemna minor* samples 100% MeOH (triangular) and 100% H_2_O (squares) extracts analyzed with single TOF-MS (violet) and QTOF-MS/MS (yellow). The confidence limit is 95%; (**c**) The S-plot of *Lemna minor* 100% MeOH and 100% H_2_O extracts with red marked molecules that represent the common ones between the two machines. Tryptophan, aspartic acid, alanine, and valine were prioritized by the PLANT-IDENT database and identified using reference standards as shown in Section 2.4 and Section 2.5.

The OPLS-DA was calculated for both systems, the 100% MeOH and 100% H_2_O extracts from *Lemna* samples as shown in Section 2.2. The OPLS-DA loading score in Figure 4a reflects that the number of the QTOF-MS/MS features (on the negative side of the first predictive component (pq1)) is higher than the features number of the single TOF-MS (on the positive side of pq1) the right side of the plot). As stated above it shows the effect of the physicochemical conditions, the matrix effect, and that each dataset was obtained with the different workflow of different software. However, the score plot of OPLS-DA explained 99.8% of the variations in the various extracts (R^2^Y (cum)) with a higher predictive value (Q^2^ (cum) = 0.891) (Figure 4b). The first components (t1) separated the different extracts according to the mass spectrometers. The statistical comparison of the data was revealed that the two sets are significantly different with a 95% confidence level. Further, the S-plot was created from the OPLS-DA model. Thereby, common metabolites can be highlighted that could be detected by the two MS in both extracts (Figure 4c). The features were with higher p(corr) values in 100% MeOH extract means that they have higher intensity in 100% MeOH extract compared to 100% H_2_O extract and vice versa. Hence, the common compounds were chosen according to p(corr) values which were small and approximately equal. The compounds marked in red represent the common metabolic profile of *Lemna minor* in both solvents using systems A and B (Figure 4c) and are listed as features in Table S4.

### 2.4. The Strategy of Lemna minor Metabolites Identification Based on the PLANT-IDENT Database (Using System B)

Data from system B was preferred due to the presence of fragment spectra from MS/MS measurements. Metabolites identification from known databases depends substantially on the following: (1) comprehensive data integration and prioritization of retention time, mass, and MS/MS fragments, and (2) considering or neglecting of MS/MS intensity information [21]. With the PI database, the uploaded analytical data can be compared and transferred via the FI platform into physicochemical parameters such as polarity and empirical formula. RT and mass are applied simultaneously as a prerequisite with MS/MS data for scoring evaluation (details in Section 3.6.2). Then, to reduce the false fragments determination, the intensity threshold is defined. The threshold differentiates the line between the signals and noise level, see Section 3.6.2.

#### 2.4.1. PLANT-IDENT Batch Searching and Scoring

(a) First, the features eluted from the HILIC column with RT ˂ 15 min were uploaded into the FI platform using the PI database (Figure S1a). The search was scored according to the mass screening, and MS/MS, each with a 50% score. Subsequently, 239 candidates were suggested according to a successive elimination approach. First, the matching features with the highest score and labeled as ‘look at’ in the platform, according to mass matching screening, and MS/MS results were considered. Then, the results were filtered and features with LogD > 0 were eliminated. After that, the chemotaxonomic filter was applied. the results were accepted, which were found in the Lemnaceae. In the end, the results with priority were considered and standards reference organized and injected for confirmation (Figure S1a). The filtred parameters decreased the number of the results from 239 to 41 potential candidates. Those metabolites were annotated and classified in the second classification level [22,23]. They could be classified to level one by confirmation with standards reference injections (see Section 2.4.2);
(b) Secondly, the RP part with RT > 15 min, thus the second part of each dataset, was uploaded. Additionally, the retention times of the reference standard mixture were uploaded to normalize the RT (Table S3 and Figure S1b). Here, the scoring of suspected compounds depends on the same parameters in addition to RTI screening [24] Each is 33% of the total score (mass screening, RTI screening, and MS/MS). One hundred and eighty-eight features were suggested as a matching candidate with the highest score and labeled as ‘look at’ in the platform. After, the adjusted logD > 0 and chemotaxonomic filter exclusively 42 candidates were considered. Those metabolites were annotated and classified in the second classification level. They could be classified to level one (i.e., an identification) by confirmation with standards reference injections (see Section 2.4.2).

#### 2.4.2. Confirmation of *Lemna minor* Metabolites Using the QTOF-MS/MS

*Lemna’s* metabolic profile was interpreted according to the following workflow from ‘precursor ion mass into molecule names’ (using data from system B, Section 2.4.1). The annotated metabolites (in classification level 2) were classified to level 1 via proven with standard reference injection [22,23]. Forty-four compounds (16 from the 41 HILIC candidates in Section 2.4.1 (a) and 28 from the 42 RPLC candidates in Section 2.4.1 (b)) could be identified with the reference standards and classified into identification level 1. These compounds are listed in Table 1 regarding their retention times. The 44 compounds have RT mostly deviations of less than 0.3 min (36 compounds below 0.3 min, five compounds below 0.5 min, one compound at 0.6 min and two compounds above 0.6 min), and a mass deviation of less than 5 ppm between the reference standard mass and the *Lemna minor* candidate feature mass. Further, MS/MS fragments of the references standards were compared with the literature. Then, the most important fragments (which were mentioned in each corresponding literature (Table 1)) were compared with unknown features fragments.

Sixteen compounds were eluted from the HILIC column and could be identified with the following reference compounds: vitexin, niacin, nicotinamide, phenylalanine, Leucine/isoleucine, tryptophan, valine, tyrosine, proline, glutamic acid, aspartic acid, di-L-alanine, 4-methoxy cinnamic acid, alanine, threonine, and serine. They have LogD < 0 at (pH = 7). In a previous study, the amino acids (phenylalanine, proline, tryptophan, alanine, tyrosine, aspartic acid, isoleucine, serine, and valine) were identified in *L. minor* using system A. The RT and mass deviations were also less than 0.3 min and 5 ppm, respectively [8,20,41]. The RT and mass deviations of both systems were less than 0.5 min. and 10 ppm. As an example for HILIC-eluting compounds the extracted ion chromatogram, *m/z* spectrum, and MS/MS spectrum of niacin (from Table 1) are compared in Figure S2 with the feature eluting at 7.8 min.

Twenty-six compounds were eluted from the RPLC column and could be identified with the following reference compounds: apigenin-6,8-di-*C*-glucopyranoside, apigenin-6-C-arabinopyranoside-8-*C*-glucopyranose, apigenin, robinetin, quercetin, luteolin, kaempferol, acacetin, orientin, isoorientin, peonidin, 6-methoxy-flavone, flavone, naringenin-7-*O*-glucoside, quercetin-3-*O*-glucoside, saponarin, 5-hydroxy-6-methoxy-flavone, luteolin-3′,7-di-*O*-glucoside, apiin, chrysoeriol, umbelliferone, norwogonin, isovitexin, tricin, galangin, and myricetin. Further, the MSMS fragments of the reference standard and the identified peaks in the *Lemna* metabolic profile were compared with the literature, as shown in (Table 1).

As an example for RPLC-eluting compounds the extracted ion chromatogram, mass spectrum, and MS/MS spectrum of apiin are compared in (Figure S3) with the feature eluting at 23.8 min.

According to chemotaxonomy criteria results, 42 compounds had previously been separated from Lemnaceae or Araceae family. According to the available literature, robinetin and norwogonin were not reported from Lemnaceae or Araceae family so far. Those two compounds could not pass the last filter of the chemotaxonomy because they are a pentahydroxy-flavone and 5,7,8-trihydroxy-flavone, respectively.

Besides, robinetin is known as 5-deoxy-myricetin. Therefore, robinetin and norwogonin reference standards were injected. In addition, luteolin, quercetin, kaempferol, apigenin, and myricetin (hydroxylated-flavone and flavanol) and their glucosides have been identified in the family before [31,42]. According to our knowledge, robinetin and norwogonin were identified for the first time in the *Lemna minor* extracts.

### 2.5. The Strategy of Lemna minor Metabolites Identification Based on the PLANT-IDENT Database (Using Common Data from Both MS Systems)

The common compounds in the metabolic profile of *Lemna minor* (systems A and B) were chosen according to p(corr) values which were marked in red (Figure 4c and Table S4). The obtained features were classified according to RT, and, prioritized using the PI database on the FI platform. The search was scored according to the mass screening (and MS/MS in system B). The PLANT-IDENT database suggested pegamine, tryptophan, aspartic acid, alanine, valine, and betaine (trimethylglycine) candidates matching features in both 100% MeOH, and 100% H_2_O extracts. They have LogD < 0 at pH = 7 (except pergamine, which was eliminated). They also have priority ‘look at’ and standards reference injected for validation except for betaine (Figure S1a). Subsequently, the filtration parameters decreased the number of the results to 5 metabolites, listed in Table S5. Those metabolites were annotated and classified in the second identification level. They could be classified into level one by confirmation with standards reference injections.

Regarding the RP part with RT > 15 min, the second part of each dataset was uploaded into the PI database in the FI platform, individually. Two features were suggested as a matching candidate in 100% MeOH, and 100% H_2_O extracts. They did not have any matches in the PI database. Those metabolites were annotated and classified in the third level.

Thus, the identification process using physicochemical parameters and the knowledge from mass spectrometric results can easily be transferred into further knowledge and to other mass spectrometers with the continuous updating of the PI database.

## 3. Materials and Methods

### 3.1. Reagents and Chemicals

LC-MS grade methanol (MeOH) and water (H_2_O; LC-MS grade) were obtained from VWR, Darmstadt, Germany. Acetonitrile (LC-MS grade) was purchased from Riedel-de-Haen (Honeywell, Seelze, Germany). 6-Amino-1,3-dimethyl-5-(formylamino)uracil, chlortoluron, famotidine, vidarabine, etilefrine, and 2,4-diamino-6-(hydroxymethyl)pteridine hydrochloride, and apigenin, monuron, chloridazon, carbetamide, metobromuron, sotalol, metconazol, vitexin, flavone (2-phenylchromone), DL-Ala-DL-Ala, nicotinic acid, nicotinamide, galangin, and acacetin were obtained from Merk, Darmstadt, Germany. Chlorbromuron was obtained from Dr. Ehrenstorfer, Augsburg, Germany. Apigenin-6-*C*-arabinoside-8-*C*-glucoside, Apigenin-6,8-di-*C*-glucoside, apiin, orientin, kaempferol, peonidin, norwogonin, luteolin-3,7-*O*-di-glucoside, 6-methoxy flavone, 4-methoxy cinnamic acid, naringenin-7-*O*-glucoside, isovitexin, tricin, quercetin-*3-O*-glucoside, saponarin, myricetin, 5-hydroxy-6-methoxy flavone, chrysoeriol, isovitexin, robinetin, and umbelliferone were kindly provided by Center of Life and Food Science Weihenstephan, Biotechnology of Natural Products, Technical University of Munich, Germany.

### 3.2. Plant Samples

*Lemna minor* L. was cultivated according to Obermeier et al. [43] with small modifications. Briefly, plants were grown at 23 °C with a photoperiod of 16–8 h and an average light intensity of 43 μmol m^−2^ s^−1^. *Lemna* fronds were subcultured every two weeks in 24 L of Steinberg medium. After harvesting, plants were shortly rinsed with distilled water, dried with lint-free tissue paper, and frozen in liquid nitrogen. Samples were kept at -80 °C until further processing. *Lemna* was kindly provided by the German Research Center for Environmental Health, Plant–Microbe Interactions, Helmholtz Centrum of Munich, Germany.

### 3.3. Sample Preparation

Five hundred milligrams of freeze-dried and milled whole *Lemna* powder were extracted with (a) 100% MeOH, (b) 50% MeOH, and (c) 100% H_2_O, respectively. The dissolved plant powders were sonicated (35 kHz, Sonorex super RK 106, Bandelin, Berlin, Germany) for 10 min at 4 °C with a 35 kHz frequency. Then, samples were centrifuged (NuWind Multi-Application Bench Top Refrigerated Centrifuge 2, NuAire, Plymouth, MN 55447, USA) at 1500 rpm/261.6× *g* for 20 min at 4 °C and the supernatants were transferred into clean glass test tubes. The extraction process was triplicated in identical experimental conditions. Finally, the extracts were evaporated to dryness (using a SpeedVac, Fischer Scientific, Göteborg, Sweden) and dissolved in 1 mL of 50% MeOH [8,41]. The extracts were diluted at 1:100 before the analysis.

For this work, separate batches of *Lemna* were used in each system analysis (A and B). In system B, two different biologicals were used, which were injected separately with a time interval of one month.

### 3.4. Instruments

#### 3.4.1. Chromatographic System for Polarity Extended Separation

*Lemna* metabolomics and reference standards were separated with LC (Agilent 1260 Infinity, 5301 Stevens Creek Blvd., Santa Clara, CA, USA) consisting of an autosampler, two binary pumps, an online degasser, a mixing chamber, a UV detector, and an isocratic pump. The LC-system was used to perform separations in a serial coupling of reversed-phase and zwitterion hydrophilic interaction liquid chromatography (HILIC). The reversed-phase separation column was a Poroshell 120 EC-C18 (50.0 × 3.0 mm, 2.7 μm; Agilent Technologies, 5301 Stevens Creek Blvd., Santa Clara, CA, USA). The HILIC column was a ZIC-HILIC column (150 × 2.1 mm, 5 μm, 200 Å; Merck Sequant, Umea, Sweden). Columns were coupled through a T-piece (Upchurch, IDEX Europe GmbH, Erlangen, Germany). The third port of the T-piece was connected to the HILIC flow pump. The injection volume was 10 μL. The mobile phase of the serial coupling (RPLC-HILIC-TOF-MS) was employed as follows: Solvent A: 10 mM of ammonium acetate in 90:10 (*v/v*) water–acetonitrile; solvent B: 10 mM of ammonium acetate in 10:90 (*v/v*) water–acetonitrile (RPLC); solvent C: acetonitrile; solvent D: water (HILIC). Further mobile phase conditions are summarized in Table 2. Further details, like the RPLC-HILIC serial coupling settings, are described in [8,44,45]. The effluent of the HILIC column was connected with another T-piece (Upchurch, IDEX Europe GmbH, Erlangen, Germany). An isocratic pump was connected to the second port of the T-piece. It provided a continuous flow of 50 µL/min acetonitrile-water (90:10, v:v) with 125 nM of purine and 6.25 nM of HP-921 MS tuning mix (Agilent Technologies, Waldbronn, Germany) for constant MS recalibration. The third port of the T-piece was connected to the ion source of the mass spectrometer.

#### 3.4.2. Mass Spectrometric Detection System A in Lab 1 (Single TOF-MS)

Samples were analyzed and detected with a ‘time-of-flight’ mass spectrometer (G6230A; Agilent Technologies, Waldbronn, Germany), equipped with the Jet Stream II ESI interface (system A). Ions were detected in positive ionization mode with a mass range of 50–2100 Dalton. The parameters were as follows: 325 °C gas temperature, 10 L/min drying gas flow, 325 °C sheath gas temperature, 7.5 L/min sheath gas flow, 45-psi nebulizer operating pressure, and 100 V fragmentor voltage.

#### 3.4.3. Mass Spectrometric Detection System B in Lab 2 (QTOF-MS/MS)

The other utilized MS system was a Triple TOF 5600 (AB SCIEX, Darmstadt, Germany) (system B) with a Duospray ion source and a TurbolonSpray ESI probe in positive ion mode. A mass range of *m/z* 65 to 2000 was scanned in full range. MS/MS was acquired in independent data acquisition mode with a mass tolerance of 50 mDa. The intensity threshold was 50 cycles per second (cps). The MS/MS data were collected in the eight parallel experiments at a collision energy of 40 ± 20 eV. The ionization parameters were set as the following: 2000 kV ion spray voltage floating (ISVF) and 650 °C turbo spray temperature. The declustering potential (DP) and collision energy (CE) were set to 46 and 40 V, respectively. The nebulizer gas (gas 1), the heater gas (gas 2), and the curtain gas were set to 44, 50, and 29 psi, respectively. All gas flows were nitrogen.

### 3.5. Internal Standards

Each sample and blank were spiked with a standards mixture of 12 substances. The mixture consists of 6-amino-1,3-dimethyl-5-(formylamino) uracil, chlortoluron, famotidine, vidarabine, etilefrine, monuron, carbetamide, metobromuron, sotalol, chlorbromuron, metconazol, and 2,4-diamino-6-(hydroxymethyl) pteridine hydrochloride to obtain a final concentration of 5 µM each. These internal standards were used to estimate the mass deviation as an absolute variation between theoretical monoisotopic mass and the mean of measured isotopic masses were calculated as (Δppm) as well as the retention time stability across all experiments. The results are summarized in (Table S2). These parameters were used to correlate the unknown features in different samples.

### 3.6. Data Collection and Preprocessing

#### 3.6.1. Single TOF-MS (System A)

Data were acquired with MassHunter Workstation LC/MS Data Acquisition software B 05.00 (Agilent Technologies, Waldbronn, Germany), and was subsequently analyzed with Profinder B.06.00 (Agilent Technologies) extracting the so-called ‘features’ by their RT, molecular mass, and their peak intensity in various extracts. This was performed in a combination of the three injections of each sample after removing the features found in the corresponding blank samples. The parameters are set to a peak filter of 1000 counts peak height, ion species to ‘positive ions’ with H^+^, Na^+^, K^+^, and NH_4_^+^, ‘charge state’ to 1, the ‘expected RT’ to ±3.00 min, and the mass to ±10 ppm. The extracted ion chromatograms (EICs) were smoothed with a Gaussian function using 9 points function width and 5000 points Gaussian width. This limits the result finally to 2000 compound groups. The detailed workflow was discussed in Wahman et al., 2022 [20].

#### 3.6.2. QTOF-MS/MS (System B)

The data were obtained with Analyst Software (version TF 1.7.1). The data evaluation consisted of peak picking, alignment, and filtering, which was done with MarkerView Software (AB SCIEX, version 1.3.1). The metabolites identification was performed using the vendor software package (Sciex OS).

Peak picking: The peak picking parameters were optimized according to the mass and retention deviations of the internal standards in all samples and blanks. The minimum and maximum retention times were 5 and 34 min, respectively. The subtraction offset was 15 scans. The noise threshold and subtraction multiplication factors were 50 and 1, respectively. Further, the minimum spectral peak width was 2 ppm (Figure 1). The noise threshold and subtraction multiplication factors have a fundamental impact on peak picking especially for the low abundance metabolites due to the matrix effect of unfractionated extracts: This was achieved using internal standards. Transformation and/or normalization of the data was also acquired in this step, which is required for univariate and multivariate statistical analysis.

Alignment and filtering: The three injections of each extract were compared and aligned. The RT variations between different injections were expected to be as good as those, observed with the internal standards. Further, mass tolerance was set to 5 ppm based on QTOF specification [46], and observations from internal standards. Features were accepted if they were found in all three injections of a sample. Then, the background was deleted (i.e., the features found in the blank corresponding to each solvent were deleted from the same extract) (Figure 1). *Lemna minor* metabolic profile investigation demands alignments of features, which were found in different extracts and the different injections of similar extracts. Further, the features were deleted, which were found in the corresponding blank.

In addition, the RTI and mass tolerance were determined according to the results of the internal standard, which led to a decrease in the number of false-positive, as well as, negative peaks (i.e., reduction of the number of features from the same metabolite). The minimum variation in the noise threshold was affected the peak extraction by increasing the number of false-positive peaks or subtracting real peaks apart.

PLANT-IDENT batch searching and scoring: After alignment and filtering, data were uploaded into the FI platform. The molecular masses of detected compounds were compared with compounds stored in the PI database, resulting in suggestions for their identification. The compound database PI containing various plant metabolites was created and implemented in the FI platform. It contains up to over 3000 plant metabolites from Lemnaceae, Poaceae, Brassicaceae, Nymphaceae families, in addition to, flavonoids, and nitrogen-containing plant metabolites. The compound’s name and plant source were collected from the available literature (articles and/or books) which was cited in the database. The chemical identifiers (smiles, LogD, InChi Key) and physicochemical properties were gathered from the PubChem and Chemspider databases. The LogD values (obtained by ChemAxon) were used to support the identification of compounds through hydrophilicity for the HILIC eluted compounds. Further, the RTI was used to support the identification of RPLC eluted compounds via hydrophobicity. The retention time of the standards mixture (Table S2) was also uploaded to normalize the RT. The normalization was performed according to the calibration curve (correlation between the RT and Log D at pH = 7) of the target analysis of the standard mixture, for more details readers referred to [24]. The mixture consists of compounds, which have affixed logD at different pHs (i.e., compounds with stable logP values) (Table S2).

The features with the highest score (the sum of different prioritization criteria e.g., mass deviation, RT, and MSMS fragments with equal weighting factors) were marked in the ‘look at’ column in the FOR-IDENT platform, which was calculated via the platform. Consequently, the marked hits fragments were compared through in-silico fragmentation MetFrag [47]. Moreover, chemotaxonomy was applied because the PLANT-IDENT contained the plant name and/or family name, which was reported to contain the corresponding metabolites.

Those features were considered and standards reference injected for validation. After injection of references standards, the important peak of each has been characterized using the available literature (Table 1). The fragments were compared with the different features using the vendor software package (Sciex OS).

#### 3.6.3. PI Batch Searching and Scoring of System A Data

The processed data from systems A were also uploaded into the FI platform. The search process was performed according to the following parameters: pH = 7, precursor ion mass deviation 5 ppm, and ion species was positive. The metabolites were considered according to their score, which is the same as system B data except for the MS/MS data. Then, the results were considered when they have negative LogD at pH = 7. In contrast, the metabolites were suspected in the RP column according to the score of the same parameter in addition to the RTI [24]. The metabolites were considered when they have positive LogD. The last step was the chemotaxonomy criteria. Each metabolite has a plant and/or family name reference according to the available literature (Figure 1).

#### 3.6.4. Classification Scheme

The different extracts were analyzed according to the previously mentioned workflow (Figure 1). Then, the plant metabolites classification was performed adopted to the scheme of Letzel et al., 2014, and Schymanski et al., 2014 and Letzel et al., 2014 [22,23], which complies five levels:

1. The identification by the reference standard;
2. The identification was performed by various criteria such as (retention time behavior, accurate mass (i.e., empirical formula), fragmentation, and chemotaxonomical criteria);
3. The identification was performed by comparison of accurate mass and fragments from different laboratories;
4. The identification is done by molecular formula or fragments comparison;
5. Mass recognition without further information; this classification scheme enhances the identification of plant metabolites in untargeted metabolomics analysis.

#### 3.6.5. Orthogonal Partial Least Square—Discriminant Analysis

The quality of features was statistically investigated from both systems (Figure 3 and Figure 4). In each experiment, *Lemna* was grown in the same condition and extracted with 100% MeOH, 50% MeOH, and 100% H_2_O extracts. The data matrix was built in the Microsoft Access Database file according to the aligned qualitative data (i.e., feature annotations/abundance as columns and sample annotations as rows), along with related metadata (i.e., plant name, extraction solvent, and mass spectrometer type). The RTs, masses, and abundances were connected to the corresponding plant, extraction solvent, and mass spectrometer type. Once the matrix was built, comprehensive statistical analyses could be performed by using the vast range of functions provided by the SIMCA software. Additionally, the mass spectrometric system was included as a secondary observation. The dataset organization and different statistical parameters were mentioned in detail in [9].

OPLS is the orthogonal modification of PLS regression analysis methods, which both are supervised statistical models on the contrary of PCA [48]. The OPLS fits well for metabolomics analysis because it can analyze a large number of variables for a small sample size. The OPLS separates the variables (X) (i.e., 8940 features) into two directions linear and orthogonal to Y. The lower number of biological replicates in this study might affect the statistical conclusion. Thus, in further studies, a community-harmonized number of more biological replicates will be analyzed to reach a power analysis > 0.8. The OPLS model can be visualized with the score plot and the loading plot, which describe the contribution of the variables. The OPLS quality is described with the cumulative variation in the matrix or cumulative variation (R^2^X; cum), the cumulative variation in the Y matrix (extracts) or R^2^-Y(cum), and the cross-validated predictive ability or Q^2^(cum) values. R^2^ is defined as a fraction of the variance explained by a component. Cross-validation of R^2^ gives Q^2^, which represents the proportion of total variation predicted by a component. Thus, the R^2^ indicates how well the variation of a variable is explained, and Q^2^ how well a variable could be predicted and estimated by cross-validation [49].

## 4. Conclusions

*Lemna’s* metabolic profile was investigated using an extended polarity liquid chromatographic system.

The applied workflow(s) was (were) investigated with statistical analysis to validate the reliability and information transfer between different mass spectrometric systems and/or laboratories. The identification strategy of untargeted data was applied using an open access PLANT-IDENT database. The identification of *Lemna* metabolites proceeded according to different filters: LogD, mass deviation, MSMS fragment comparison, and chemotaxonomy filter. After prioritization, compounds were identified using reference standards leading to compounds of identification level 1. Further, from the statistical analysis, the *L. minor* metabolic profile has common features between the single TOF and QTOF-MS. The different extracts could be significantly discriminated in both MS, which could be used for further generations of *Lemna* metabolite measurements.

Thus, the untargeted plant metabolomics research will be enhanced via utilizing the workflow combined with the PLANT-IDENT database and with continuous updating of the database.

## Figures and Tables

**Table 1:** Compounds identified in *Lemna minor* metabolic profile via PLANT-IDENT database. Retention time (RT) of standards (S), measured features (M), and the RT deviation, as well as the mass of standards (S), measured features (M), and the deviation between them, were listed. The mean fragments of standards and measured were compared with the literature and listed with the references; (*) means compounds detected in system A (single TOF-MS).

Compound Name	RT (S) [Min]	RT (M) [Min]	ΔRT [Min]	Mass (S) [Da]	Mass (M) [Da]	Δppm	MSMS Fragments	References
Vitexin	7.5	7.3	0.2	433.1133	433.1129	0.8	433;415;397;379;337;313; 283	[25]
Niacin *	7.6	7.8	−0.2	124.0394	124.0393	0.7	124;96;80;78	[26]
Nicotinamide	7.8	7.6	0.1	123.0554	123.0553	0.8	123;106;80;78	[27]
Phenylalanine *	11.0	11.1	−0.1	166.0866	166.08627	2.0	120;103;77	MassBank of North America (MoNA)
Leucine/Isoleucine *	11.2	11.2	−0.1	132.1018	132.1020	−2.0	86;69;44;30	(MoNA)
Tryptophan *	11.7	11.7	0.0	205.0973	205.0970	1.8	188;146;144	(MoNA)
Valine *	12.1	11.9	0.1	118.0863	118.0862	0.8	72;71;55	(MoNA)
Tyrosine *	12.3	12.2	0.1	182.0811	182.0810	1.9	136;123;119	(MoNA)
Proline *	12.4	12.4	0.0	116.0705	116.0707	0.3	70;68;43	(MoNA)
Glutamic acid *	12.5	12.6	−0.1	147.0434	147.0430	3.0	130;102;84	(MoNA)
Aspartic acid *	12.7	12.7	0.0	134.0447	134.0447	−0.0	134;115	(MoNA)
Di-L-Alanine	12.7	12.8	−0.1	161.0928	161.0920	4.9	161;115;90	(MoNA)
4-Methoxy cinnamic acid	13.4	13.1	0.3	179.0706	179.0708	−0.9	147;137	[28]
Alanine*	13.4	13.2	0.2	90.0550	90.0548	2.1	44;28	(MoNA)
Threonine*	13.6	13.4	0.2	120.0656	120.0653	2.8	73;56	(MoNA)
Serine *	14.0	13.8	0.2	106.0500	116.0499	0.9	60;42;43	(MoNA)
Apigenin-6,8-di-*C*-glucopyranoside *	15.8	15.7	0.1	595.1659	595.1658	0.2	595; 383	[29]
Robinetin	15.8	15.9	−0.1	303.0494	303.0493	0.3	285;267;147	(MoNA)
Apigenin-6-*C*-arabopyranoside-8- *C*-glucopyranose	23.4	23.3	0.2	565.1550	565.1557	−1.2	565;547;379;337;325;295;121	[30]
Luteolin-3′,7-di-*O*-glucoside	23.8	23.6	0.3	611.1640	611.1622	2.8	611;449;287	(MoNA)
Saponarin	23.8	24.0	−0.2	595.1638	595.1663	−4.2	433;415;397;367;337;283;271	[31]
Isoorientin	23.8	23.6	0.2	449.1085	449.1095	−2.1	449;329;299;165	[32]
Isovitexin	24.1	23.9	0.2	433.1125	433.1134	−2.0	313;295;284;283;267	[25]
Norwogonin	24.2	24.0	0.2	271.0604	271.0599	1.8	271;253;241;225	[33]
Quercetin-3-*O*-glucoside	24.2	24.3	−0.1	465.1018	465.1022	−0.7	465; 303	[34]
Apiin	24.6	23.8	0.9	565.1566	565.1559	1.3	433;313	[35]
Umbelliferone	24.7	24.4	0.2	163.0396	163.0391	2.9	135;107	[28]
Quercetin	24.8	24.9	−0.1	303.0549	303.0544	1.7	303;285;257;229;165	[36]
Luteolin	24.8	24.6	0.2	287.0562	287.0557	1.6	287;269;241;153	[36]
Naringenin-7-*O*-glucoside	25.0	24.1	0.9	435.1298	435.1285	2.9	435;273	[37]
Myricetin	25.1	25.1	0.0	319.0440	319.0453	4.0	301;283;265;111	[38]
Orientin	25.1	25.2	−0.1	449.1123	449.1134	−2.6	449; 329	[29]
Peonidin	25.6	25.2	0.4	302.0785	302.0792	−2.4	302;283;197	(MoNA)
Chrysoeriol	26.9	26.8	0.1	301.0731	301.0722	2.9	286;121	[39]
Tricin	26.8	26.3	0.6	331.0811	331.0796	4.7	331;315	[5]
Apigenin	26.8	26.7	0.1	271.0603	271.0604	−0.6	271;253;153	[36]
Acacetin	28.8	29.1	−0.3	285.0759	285.0760	−0.4	285;242;153	[39]
Kaempferol	29.0	29.1	−0.1	287.0531	287.0540	−3.1	287;269;231;165;153;133	[36]
Galangin	29.4	29.4	−0.1	271.0602	271.0608	2.3	271;253	[40]
Flavone (2-Phenylchromone)	29.9	29.6	0.2	223.0756	223.0748	3.6	223;178;152;121	(MoNA)
6-Methoxyflavone	30.4	30.6	−0.2	253.0879	253.0881	−0.7	253; 238; 210	NIST
5-Hydroxy-6-Methoxyflavone	31.3	31.1	0.1	269.0823	269.0819	1.3	269;254;104	(MoNA)

https://mona.fiehnlab.ucdavis.e accessed on 30 November 2021.

**Table 2:** Mobile phase condition of RPLC-HILIC-TOF-MS.

Binary Pump 1				Binary Pump 2			
Time (min)	Flow Rate (mL/min)	A%	B%	Time (min)	Flow Rate (mL/min)	C%	D%
1	0.05	100	0	0	0.4	100	0
7	0.05	100	0	6	0.4	100	0
12	0.05	50	50	13	0.4	60	40
13	0.1	50	50	32	0.4	60	40
22	0.1	0	100	33	0.8	100	0
32	0.1	0	100	53	0.8	100	0
33	0.1	100	0	54	0.4	100	0
53	0.1	100	0	58	0.4	100	0
54	0.05	100	0				
58	0.05	100	0				

## Data Availability

The data presented in this study are available on request from the corresponding author. The data are not publicly available due to contiouos work on it.

## Supplementary Materials

The following are available online at https://www.mdpi.com/article/10.3390/metabo11120832/s1, Figure S1: (**a**) Search for the feature masses, which were separated with the HILIC column from the 100% MeOH extract; (**b**) RTI handling according to the pH of the compounds as the prioritization tool in PLANT-IDENT database in the FOR-IDENT platform for more information readers referred to the FOR-IDENT manual (https://water.for-ident.org/#!home (accessed on 29 November 2021)), Figure S2: (**a**) Extracted ion chromatogram (EIC) of niacin shows intensity in counts per second (cps), appears on the *y*-axis, while RT appears on the *x*-axis; (**b**) mass spectrum of niacin represents the masses and intensity of the ions with particular mass-to-charge (*m/z*) values in Daltons; (**c**) MS/MS spectrum of niacin shows the fragments and relative intensity (%), Figure S3: (**a**) Extracted ion chromatogram (EIC) of apiin shows intensity in counts per second (cps), appears on the *y*-axis, while RT appears on the *x*-axis; (**b**) mass spectrum of apiin represents the masses and intensity of the ions with particular mass-to-charge (*m/z*) values in Daltons; (**c**) MS/MS spectrum of apiin shows the fragments and relative intensity (%), Table S1: The compounds of *Lemna minor* metabolic profile identified in the different samples which have been measured with Agilent and Sciex machines, Table S2: The internal standards mean monoisotopic mass in Daltons, mean mass standard deviation (SD), mean RT in minutes, mean RT standard deviation (SD), and relative standard deviation (RSD) were listed, Table S3: The RTI calibration compounds mean monoisotopic mass of in Daltons, mean mass standard deviation (SD), mean RT in minutes, mean RT standard deviation (SD), and relative standard deviation (RSD) were listed, Table S4: The list of marked compounds in the S-plot, Table S5: The proposed compounds in the HILIC part of *Lemna minor* metabolomics by the PLANT-IDENT database.
