# Supplementary material for: Untargeted Analysis of Lemna minor Metabolites: Workflow and Prioritization Strategy Comparing Highly Confident Features between Different Mass Spectrometers
Source: Metabolites. 2021 Dec 2;11(12):832. doi: 10.3390/metabo11120832 (PMC8706044; doi:10.3390/metabo11120832)
Supplement: Supplementary file 1 [file metabolites-11-00832-s001.zip › metabolites-1409936-supplementary.pdf]

Untargeted analysis of *Lemna minor* metabolites: Workflow and prioritization strategy comparing highly confident features between different mass spectrometers

Rofida Wahman[1,2], Stefan Moser[3], Stefan Bieber [4], Catarina Cruzeiro[5], Peter Schröder[5], August Gilg[6], Frank Lesske[6], and Thomas Letzel [1,4*]

1 Chair of Urban Water Systems Engineering, Technical University of Munich, Am Coulombwall 3, 85748 Garching, Germany.
2 Pharmacognosy Department, Faculty of Pharmacy, Assiut University, Assiut, Arab Republic of Egypt/Egypt.
3 Stefan Moser Process Optimization, Weberweg 3, D-83131 Nußdorf am Inn, Germany
4 Analytisches Forschungsinstitut für Non-Target Screening GmbH (AFIN-TS), Am Mittleren Moos 48, 86167 Augsburg, Germany.
5 German Research Center for Environmental Health, Research Unit Comparative Microbiome Analysis, Helmholtz Centrum Munich, Ingolstädter Strasse 1, 85764 Neuherberg, Germany
6 University of Applied Sciences, Am Hofgarten 4, 85354 Freising, Weihenstephan, Germany

*****Correspondence: t.letzel@afin-ts.de

Table S1: The compounds of *Lemna minor* metabolic profile identified in the different samples which have been measured with System A and B

**Compound name**	**System A**		**System B**	
	RT (Min)_	Mass (Da)	RT (Min)_	Mass (Da)
**Tyrosine**	12.2	181.08	12.0	181.08
**Aspartic acid**	12.9	133.04	12.7	133.04
**Phenylalanine**	10.7	165.08	11.1	165.09
**Tryptophan**	11.4	204.10	11.7	204.10
**Valine**	11.6	117.08	11.9	117.09
**L-alanine**	13.1	89.05	13.2	89.05
**Serine**	13.5	105.04	13.8	105.05
**L-isoleucine**	10.9	131.10	11.2	131.10
**L-proline**	12.4	115.07	12.4	115.07
**Niacin**	7.6	123.04	7.8	123.04
**Apigenin-6,8-diglucopyranosid**	15.5	594.15	15.7	594.17

Table S2: The internal standards mean monoisotopic mass in Dalton, mean mass standard deviation (SD), mean retention times (RT; min), mean RT standard deviation (SD), and relative standard deviation (RSD) were listed.

Compound Name	Mean Mass (Da)	SD (Da)	RT Mean (Min)	SD RT (Min)	%RSD
**2,4-Diamino-6-(hydroxymethyl)pteridine**	193.0837	0.0003	8.92	0.09	1.04
**6-Amino-1,3dimethyl-5-(formylamino)uracil**	199.0831	0.0004	6.53	0.09	1.31
**Carbetamid**	237.1239	0.0004	26.18	0.03	0.09
**Chloridazon**	222.0437	0.0006	24.9	0.03	0.12
**Chlorotoluron**	213.0798	0.0007	27.66	0.03	0.10
**Etilefrine**	182.1181	0.0004	11.71	0.05	0.42
**Famotidine**	338.0530	0.0007	15.44	0.15	0.94
**Metconazol**	320.1539	0.0008	30.98	0.06	0.18
**Metobromuron**	259.0082	0.0007	28.60	0.03	0.11
**Monuron**	199.0639	0.0006	26.65	0.02	0.09
**Sotalol**	273.1276	0.0007	15.09	0.23	1.51
**Vidarabine**	268.1048	0.0006	9.69	0.11	1.17

Table S3: The RTI calibration compounds mean monoisotopic mass of in Dalton, mean mass standard deviation (SD), mean RT (min), mean RT standard deviation (SD), and relative standard deviation (RSD) were listed.

Compound Name	Mean Mass (Da)	SD (Da)	RT Mean (Min)	SD RT (Min)	%RSD
**Metformin**	129.1007	0.001	14.26	0.09	0.7
**Monuron**	199.0639	0.0006	26.65	0.02	0.09
**Carbetamide**	237.1239	0.0004	26.18	0.03	0.09
**Chloridazon**	222.0437	0.0006	24.9	0.03	0.12
**Chlorbromuron**	291.9614	0	27.55	0.37	1.3
**Metconazol**	320.1539	0.0008	30.98	0.06	0.18
**Metobromuron**	259.0082	0.0007	28.60	0.03	0.11

Table S4: The list of red-marked compounds in the S-plot of Figure 4c.

Primary ID	Module p[1]	Module p(corr)[1]	Primary ID	Module p[1]	Module p(corr)[1]
602.11@25.5	0.01187	0.329627	131.1@10.9	0.009783	0.501853
327.22@10.3	0.011676	0.324272	105.04@13.5	0.008167	0.412485
245.16@13.1	0.011446	0.31758	89.05@13.1	0.007009	0.357503
133.04@12.9	0.009959	0.520567	167.08@07.9	0.006478	0.180549
123.04@7.6	0.010238	0.527519	181.08@12.2	0.005688	0.278633
204.1@11.4	0.010192	0.52508	211.16@14.0	0.005578	0.154208
117.09@11.6	0.010191	0.528166	115.07@12.4	0.005334	0.293226
165.08@10.9	0.01018	0.528748	273.19@12.4	0.005309	0.147462
283.22@14.0	0.009988	0.276824	287.18@11.9	0.003642	0.102415
347.27@24.8	0.009973	0.277353	509.32@14.7	0.000496	0.012598

Table S5: The proposed compounds in the HILIC part of *Lemna minor* metabolic profile by the PLANT-IDENT database, which were detected in system A and B data

ID	Best match	Weighted Score	Mass Screening Score	Target RT	Target mass	Δ mass	Name	Elemental formula	logD at pH=7	Category
1	false	0.45	0.45	11.73	204.0899	-0.0001	Tryptophan	C11H12N2O2	-1.09	Lemnaceae or Araceae
2	true	0.35	0.35	13.13	133.0373	-0.0002	Aspartic acid	C4H7NO4	-5.37	Brassicaceae
3	true	0.05	0.05	12.69	89.0476	-0.0004	Alanine	C3H7NO2	-2.84	Lemnaceae or Araceae
4	true	0.41	0.41	11.59	117.0789	-0.0001	Valine	C5H11NO2	-1.95	Nymphaeaceae
5	true	0.41	0.41	11.59	117.0789	-0.0001	Betaine	C5H11NO2	-3.72	Bocconia frutescens

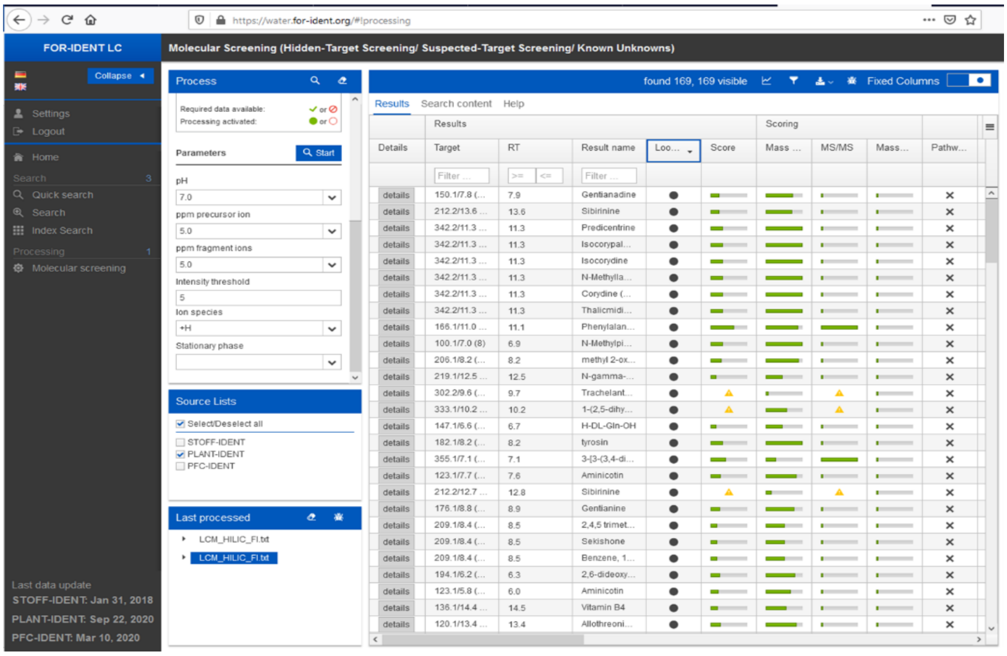

(a)

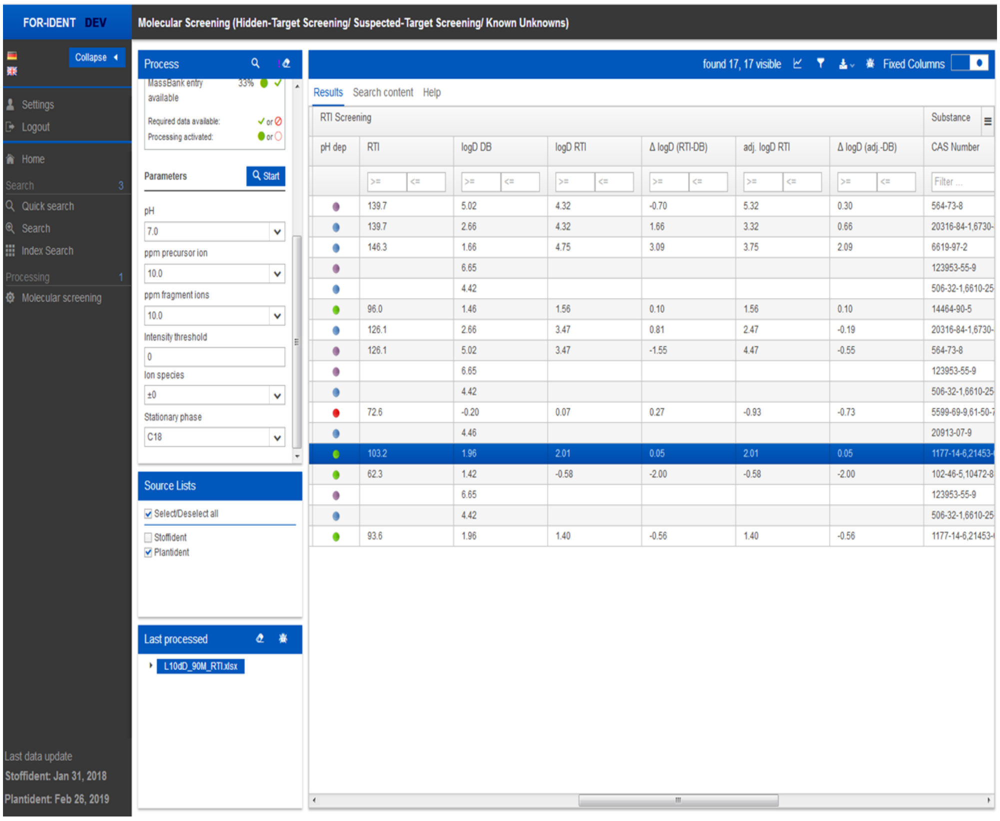

(b)

Figure S1: (a) Search for the feature masses, which were separated with the HILIC column from the 100% MeOH extract; (b) RTI handling according to the pH of the compounds as the prioritization tool in PLANT-IDENT database in the FOR-IDENT platform for more information readers referred to the FOR-IDENT manual (https://water.for-ident.org/#!home).

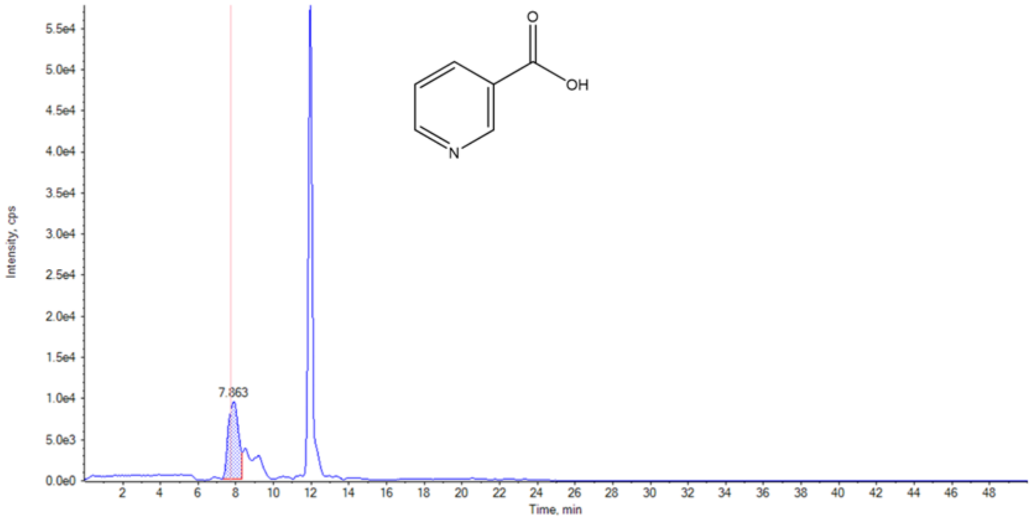
7.863
Intensity, cps
Time, min
O
OH
N

(a)

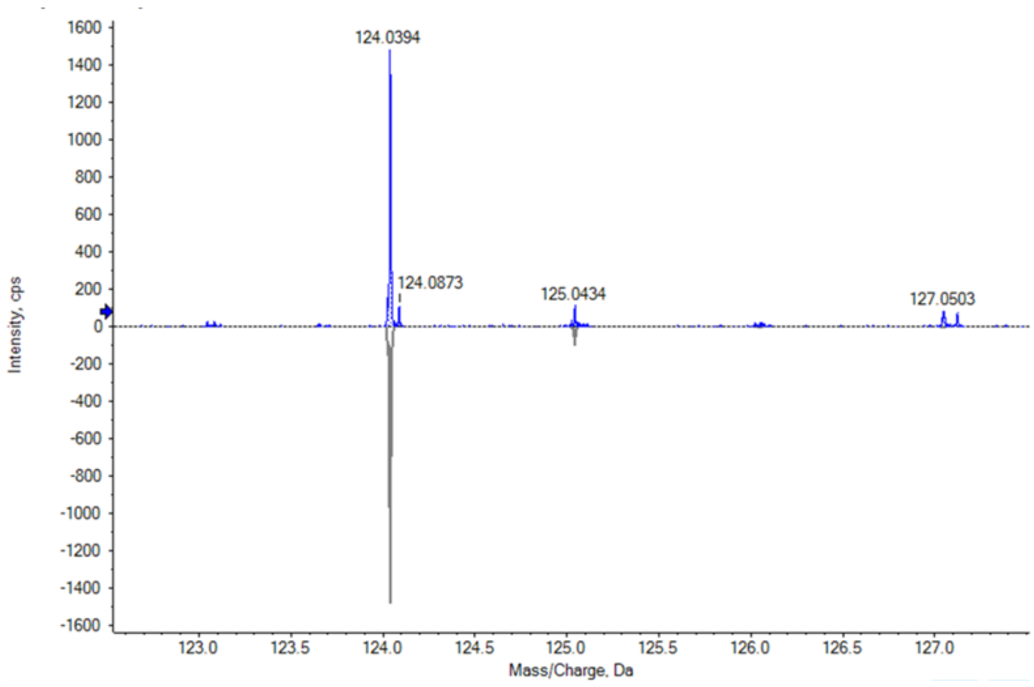
124.0394
124.0873
125.0434
127.0503
Intensity, cps
Mass/Charge, Da

(b)

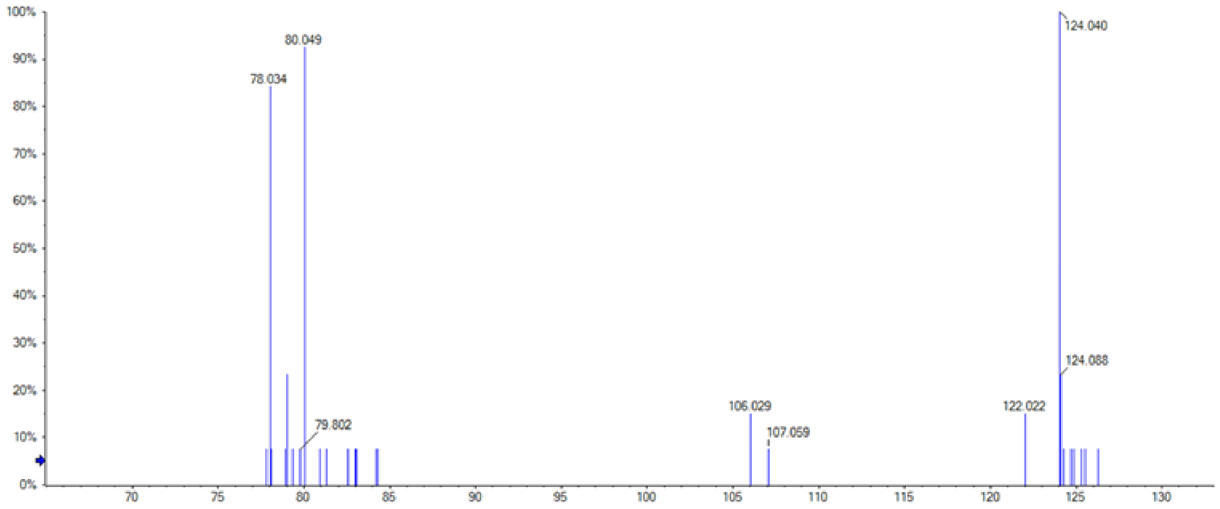

(c)

Figure S2: (a)-Extracted ion chromatogram (EIC) of niacin shows intensity (1.0 e4) in counts per second (cps), appears on the y-axis, while RT appears on the x-axis; (b)-Mass Spectrum of niacin represents the masses and intensity of the ions with particular mass-to-charge (m/z) values in Daltons; (c) - MS/MS spectrum of niacin shows the fragments and relative intensity (%).

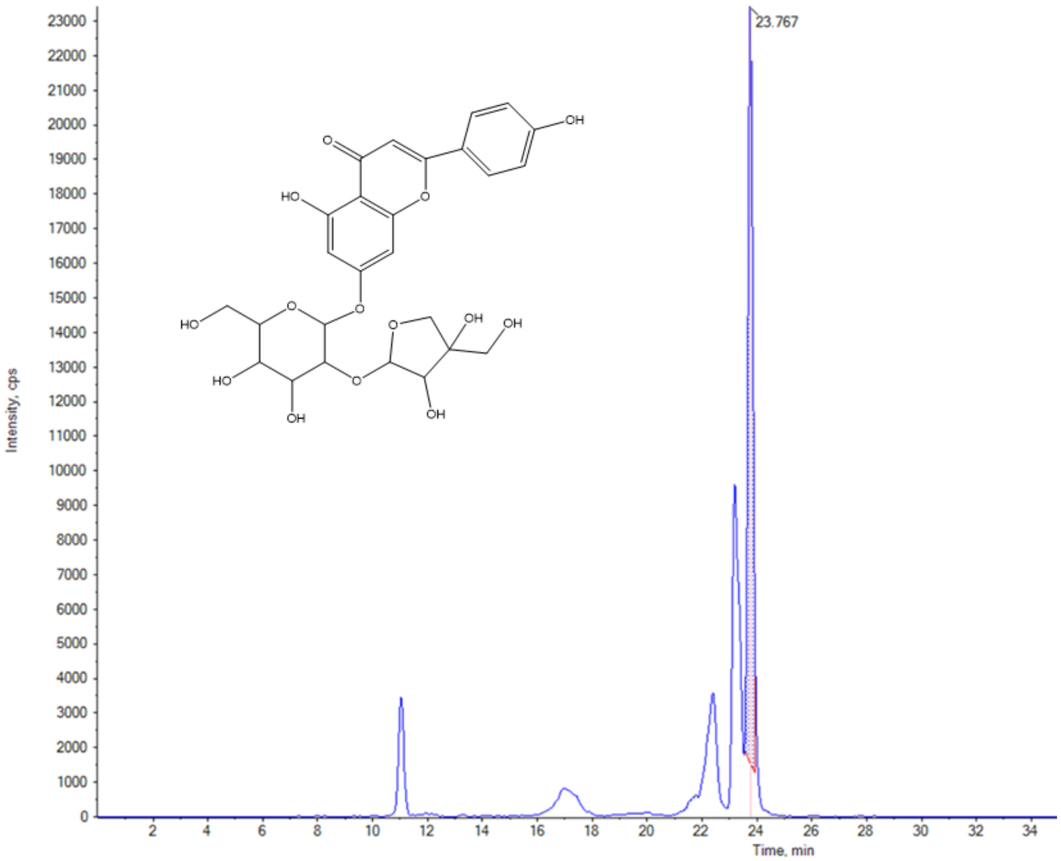
23.767
Intensity, cps
Time, min
HO
OH
O

(a)

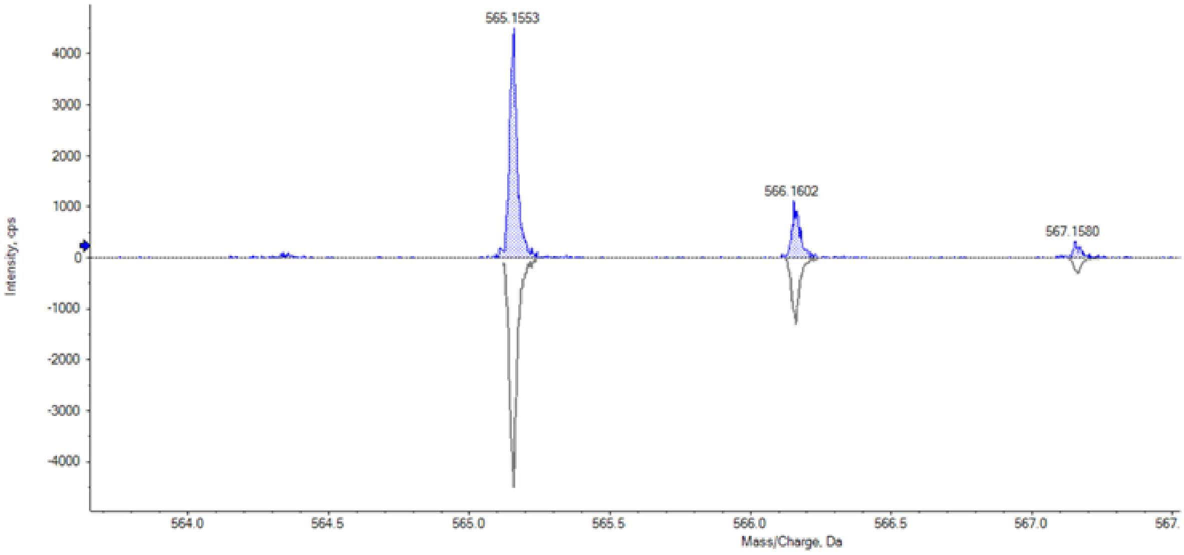
565.1553
566.1602
567.1580
Intensity, cps
Mass/Charge, Da

(b)

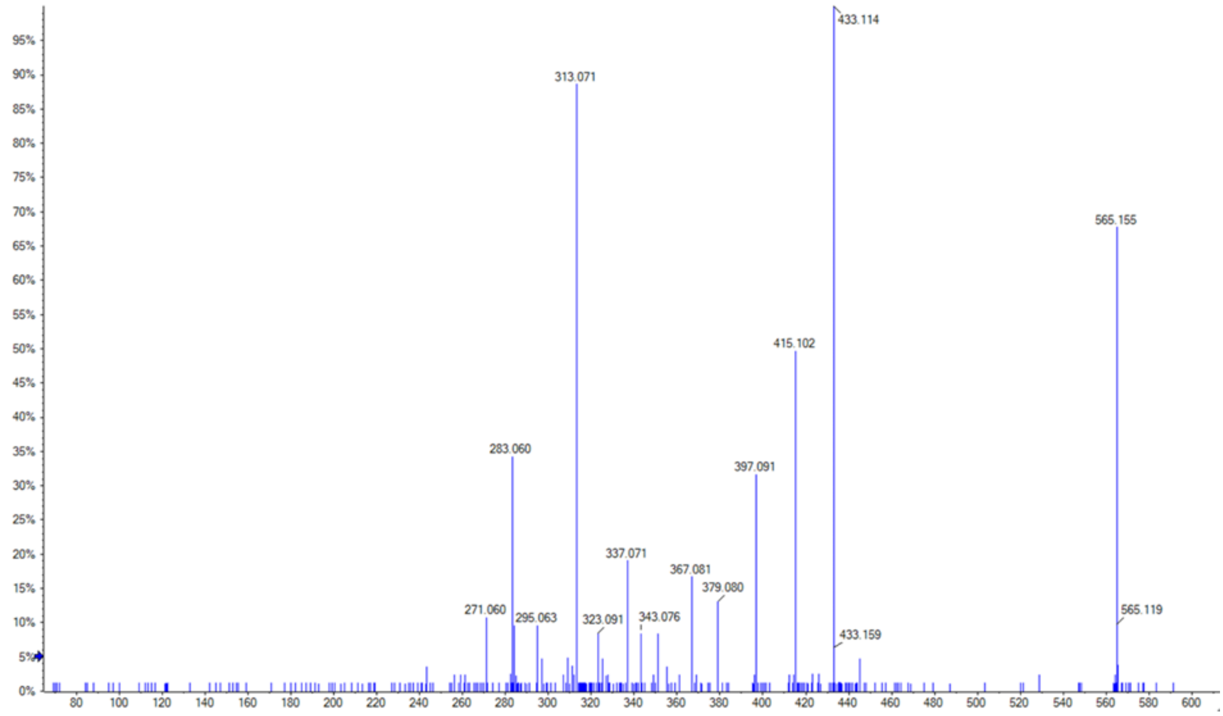

(c)

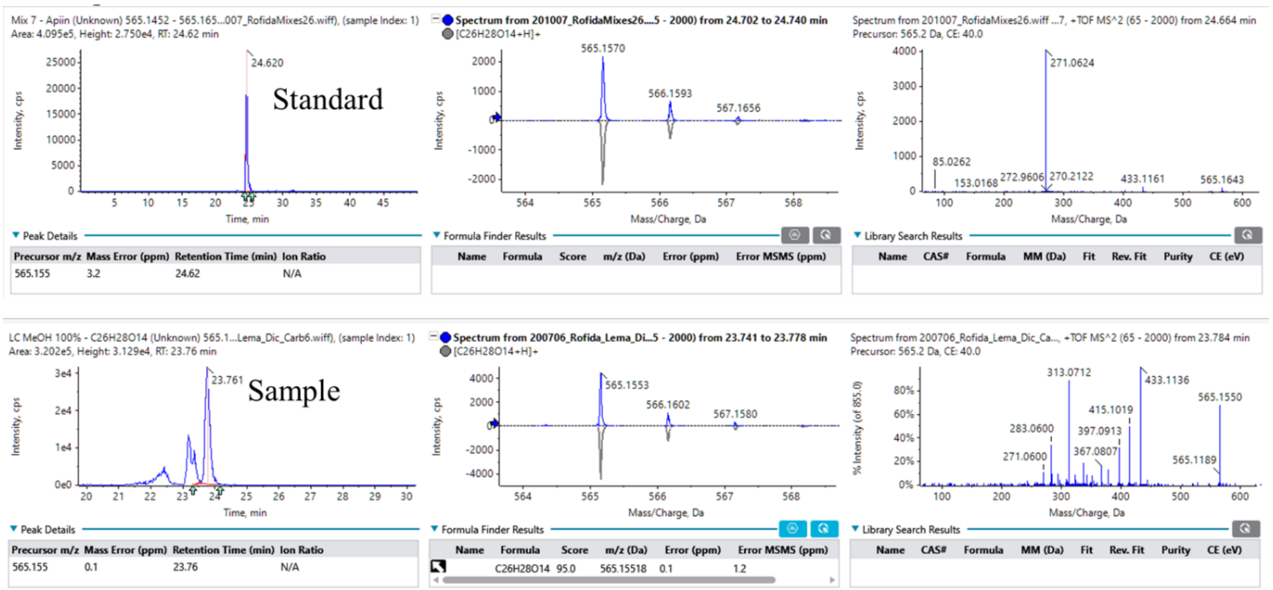

(d)

Figure S3: (a)-Extracted ion chromatogram (EIC) of apiin shows intensity in counts per second (cps), appears on the y-axis, while RT appears on the x-axis; (b)-Mass Spectrum of apiin represents the masses and intensity of the ions with particular mass-to-charge (m/z) values in Daltons; (c)- MS/MS spectrum of apiin shows the fragments and relative intensity (%); (d)- The EIC, mass Spectrum, and the MS/MS spectrum of apiin standard and sample.
